# Outlines of Materia Medica and Pharmacology

**Published:** 1896-06

**Authors:** 


Outlines of Materia Medica and Pharmacology. A Text-book for Students. By H. M. Bracken, M. D., Professor of Materia Medica, Therapeutics and Clinical Medicine, University of Minnesota. Octavo, pp. 383. Price, $2.75. Philadelphia: P. Blakiston, Son & Co., 1012 Walnut street. 1895.

The author states in his preface that the volume was designed as an aid to the student in materia medica. As such it is to be recommended. The classification is good and the book gives in a systematic way the principal facts that the student should know about each drug. The newer preparations have received due attention. The statement of the author that inversion of the body in dangerous chloroform narcosis is to be avoided is rather surprising, as it is at variance with the general teaching, for undoubtedly a number of lives have been saved by this method. The book is of little use to the practising physician, as the therapeutical details are too meager, but it is an efficient aid to the student. H. K.

